# Strain dependent effects of conditioned fear in adult C57Bl/6 and Balb/C mice following postnatal exposure to chlorpyrifos: relation to expression of brain acetylcholinesterase mRNA

**DOI:** 10.3389/fnbeh.2015.00110

**Published:** 2015-04-29

**Authors:** Sarit Oriel, Ora Kofman

**Affiliations:** Department of Psychology and Zlotowski Center for Neuroscience, Ben-Gurion University of the NegevBeer-Sheva, Israel

**Keywords:** organophosphate pesticides, chlorpyrifos, acetylcholinesterase, fear conditioning, active avoidance, depression

## Abstract

Following reports of emotional psychopathology in children and adults exposed to organophosphates, the effects of postnatal chlorpyrifos (CPF) on fear-conditioning and depression-like behaviors were tested in adult mice. Concomitant changes in expression of mRNA for synaptic and soluble splice variants of acetylcholinesterase (AChE) were examined in mouse pups and adults of the Balb/C and C57Bl/6 (B6) strains, which differ in their behavioral and hormonal stress response. Mice were injected subcutaneously with 1 mg/kg CPF on postnatal days 4–10 and tested as adults for conditioned fear, sucrose preference, and forced swim. Acetylcholinesterase activity was assessed in the brains of pups on the first and last day of treatment. Expression of soluble and synaptic AChE mRNA was assessed in brains of treated pups and fear-conditioned adults using real-time PCR. Adult Balb/C mice exposed postnatally to CPF showed exacerbated fear-conditioning and impaired active avoidance. Adult B6 mice exposed postnatally to CPF showed a more specific fear response to tones and less freezing in the inter-tone intervals, in contrast to the vehicle-pretreated mice. Chlorpyrifos also attenuated sweet preference and enhanced climbing in the forced swim test. Chlorpyrifos-treated mice had increased expression of both synaptic and readthrough AChE transcripts in the hippocampus of Balb/C mice and decreased expression in the amygdala following fear-conditioning. In conclusion, postnatal CPF had long-term effects on fear and depression, as well as on expression of AChE mRNA. These changes may be related to alteration in the interaction between hippocampus and amygdala in regulating negative emotions.

## Introduction

Extensive use of organophosphate pesticides (OPs), including chlorpyrifos (CPF), in agriculture is a subject of concern because of potential neurodevelopmental deficits following low level exposure. Exposure to OPs is ubiquitous from gestation to adulthood. OP residues have been found in rural and urban areas (Eskenazi et al., 2010; Engel et al., 2011) and children under age 5 were estimated to inhale 2 ng/kg/day and ingest close to 5 ng/kg/day (Heudorf et al., 2004; Lu et al., 2008). Longitudinal studies in the USA indicated an association between gestational exposure to OPs and lower scores on IQ tests, attention deficits, impaired neurocognitive functions (Rauh et al., 2006, 2011; Marks et al., 2010; Bouchard et al., 2011; Engel et al., 2011), altered frontal lobe structure (Rauh et al., 2012), and increased signs of pervasive developmental disorder (PDD) and social dysfunction in specific populations of children (Engel et al., 2007; Eskenazi et al., 2007; Furlong et al., 2014). Prenatal and postnatal exposure to CPF were found to affect hippocampal-dependent learning (Icenogle et al., 2004), anxiety in the elevated plus maze (EPM), anhedonia (Aldridge et al., 2005a; Ricceri et al., 2006), maternal behavior, ultrasonic vocalizations (Venerosi et al., 2009, 2010), and locomotor activity (Dam et al., 2000) in rodents. OP exposure was also cited as a risk factor for depression, anxiety and impulsivity in children, adolescents, and adults exposed to relatively high doses following home fumigation or agricultural labor (Levin et al., 1976; Stallones and Beseler, 2002; Delgado et al., 2004; Ruckart et al., 2004; Rohlman et al., 2005). Exposure to OPs during development could enhance the risk for emergence of affective disorders in later life, contributing to the “silent pandemic” of pesticide exposure (Grandjean and Landrigan, 2006). Preclinical studies showed that early exposure to OPs showed inconsistent patterns in rodent and fish assays of anxiety. A reduction in the EPM anxiety assay was reported following perinatal exposure to CPF (Aldridge et al., 2005a; Ricceri et al., 2006) or diazinon (Roegge et al., 2008) but increased anxiety was reported in female mice after extended gestational and postnatal CPF exposure (Braquenier et al., 2010). Similarly CPF-induced changes in fish revealed either increased (Eddins et al., 2010) or decreased anxiety (Richendrfer et al., 2012). Therefore, in this study we specifically focused on cued conditioned fear responses which are used to model phobic and trauma-related psychopathology.

In order to elucidate the mechanism of action underlying the long-term effects of CPF, non-catalytic effects of acetylcholinesterase AChE were examined. AChE contributes to synaptic plasticity in many brain areas in response to stress or OP exposure and is involved in cognitive and emotional behaviors (Soreq and Seidman, 2001). Post-transcriptional non-catalytic effects of AChE were examined by measuring the expression of the synaptic tetramer (AChE-S) and the soluble rare readthrough monomer (AChE-R) (Soreq and Seidman, 2001; Jameson et al., 2007). Although an association between upregulation of the rare readthrough AChE-R splice variant and OP exposure has been reported in some studies (Meshorer et al., 2002; Perrier et al., 2005), others found these effects to be common to both isoforms when the OPs were administered during early development (Jameson et al., 2007; Oriel et al., 2014). Stress-induced alterations in the expression of AChE splice variants were found in the adult brain (Dori et al., 2011; Shaltiel et al., 2013), leading us to postulate that early exposure to CPF could result in long-term changes in expression of AChE transcripts.

In this study, the immediate and protracted effects of developmental sub-toxic doses of CPF on behavior and modulation of AChE splicing were investigated in two strains of mice which differ markedly in their emotional behavior, Balb/C and C57BL/6J (B6). Balb/C mice were reported to be more anxious than B6 in some, but not all tests of anxiety such as the EPM and light/dark box (Griebel et al., 2000; Lepicard et al., 2000; Anisman et al., 2001; Ducottet and Belzung, 2005), showed more immobility (Millstein and Holmes, 2007; Livneh et al., 2010) and greater corticosterone elevations (Anisman et al., 2001) in response to the forced swim test (FST) compared to B6 mice. We hypothesized that: (1) postnatal CPF would exacerbate conditioned fear and depression-like behaviors in adult mice and that (2) AChE-R and AChE-S mRNA expression would be enhanced in pup forebrains and in brains of adult mice that had undergone CPF treatment postnatally.

## Materials and methods

### Animals

**Figure 1:**
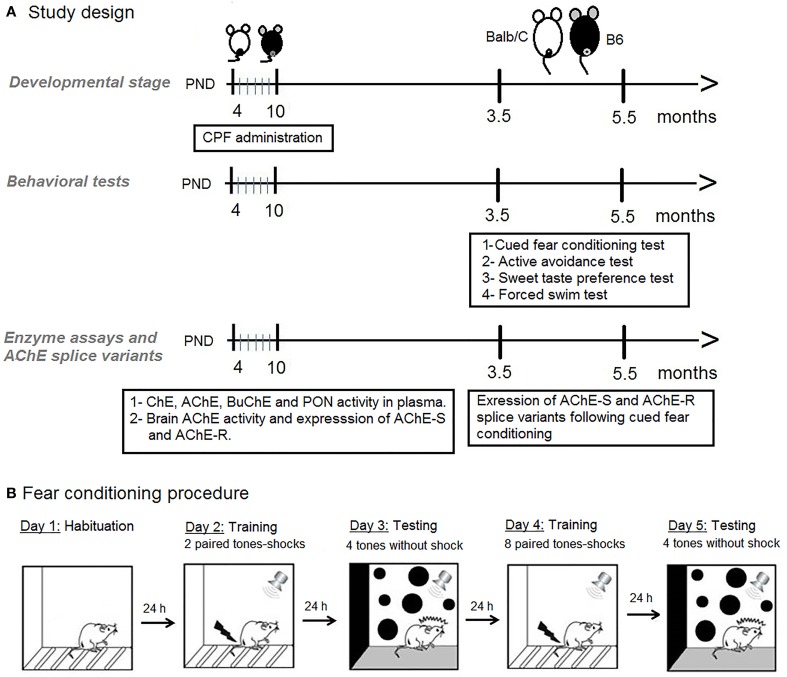
**(A)** Schematic diagram of the study design. **(B)** Schematic presentation of the fear conditioning procedure.

Balb/C and C57Bl6/J (B6) males were bred in our laboratory from dams and sires purchased from Harlan, Israel. Mice were housed with a reversed 12/12 light cycle (lights on 8 p.m.). On postnatal day (PND)-4 (day of birth is designated 0), mouse pups were randomly assigned to treatment groups and subcutaneously (s.c.) injected daily in the dark phase with chlorpyrifos (CPF, ChemService Inc., ≤98% purity) (1 mg/10 ml/kg body weight) or an equivalent volume of the vehicle (30% solution of dimethyl sulfoxide (DMSO, Sigma-Aldrich, Israel in 0.9% saline) (Aldridge et al., 2003) on PND 4–10. A no treatment (NT) group was weighed and handled to control for the effect of injection stress. No overt sign of cholinergic toxicity, such as diarrhea and lacrimation were observed. Litters were weaned on PND-28 and housed with same sex littermates until testing (3.5–5.5 months). All procedures were approved by the Institutional Committee for the Ethical Care and Use of the Animals. One male from each litter was used for each of the fear-conditioning and active avoidance test. Sweet preference and the forced swim test were tested in that order on different naïve mice, one per litter. The behavioral tests were conducted in the order described below as depicted in Figure 1.

### AChE activity in pup brains

AChE activity was analyzed on PN4 and PND-10, 2 h after the injection corresponding to the time of peak inhibition (Dam et al., 2000). Two hours post-treatment at PND-4 and -10, mouse pups were anesthetized with isofluorane, quickly decapitated and the brains dissected on ice into forebrain, midbrain, and hindbrain. Brain samples were frozen at −80°C until analyzed. Preparation of brain homogenates in each brain region was conducted using a modified spectrophotometric method (Ellman et al., 1961). Brain regions were thawed and homogenized in ice-cold sodium phosphate buffer pH = 7.4 containing 1% triton X100 (Chiappa et al., 1995; Dam et al., 2000; Das et al., 2000). The protein concentration for each brain homogenate sample was carried out using Pierce BCA Protein Assay (Ornat-Israel) with bovine serum albumin (Sigma-Aldrich) to allow estimation of μg/ml protein in the sample (Zimmermann et al., 2008). AChE activity was determined by triplicate using AcThCh (acetylthiocholine) or both Iso-OMPA (tetraisopropylpyrophosphoramide is a specific BuChE inhibitor) and AcThCh (Sigma-Aldrich, Israel) as the substrate and by measuring the colorimetric change at a wavelength of 412 nm.

Readings at ε 412 nm were repeated at 2-min intervals for 20 min. Non-enzymatic breakdown of substrate was subtracted from the total rate of hydrolysis. Enzyme activities were calculated using the ε 412 for 5-thio-2-nitrobenzoate, 13,600 M/cm. A unit of AChE activity was defined as 1 μmol of substrate hydrolyzed/min. The assay for each sample was run in triplicate and each sample was performed thrice.

### Fear conditioning

#### Apparatus

Fear conditioning tests were conducted in a two-chambered active avoidance apparatus (20 × 20 × 24 cm) (model LE 916/918, Panlab, Spain) which had 3 black Perspex walls and a transparent front wall. The floor was made from stainless steel bars through which footshock was delivered. Cued fear testing occurred in the second unfamiliar chamber which was altered by changing the surfaces of the walls, floor and adding a drop of vanilla scent (Curzon et al., 2009), such that cue was more salient than the context in order to avoid contextual fear generalization.

#### Pavlovian fear conditioning and testing

Adult mice (4 months) from the six treatment groups were studied in a factorial design 2 × 3: 2 strains (Balb/C, B6) × 3 treatments (NT, Vehicle, CPF). The cued fear-conditioning procedure included different phases across 3 days as described in Figure 1B and detailed below.

Day 1-Habituation: Each mouse was placed in the test chamber and allowed to explore for 30 min.
Day 2-Brief training: After a 4 min habituation period, each mouse was exposed to 2 pairings of an auditory cue (CS: 20 sec at 80 dB) co-terminated with a 0.4 mA footshock US during the last 2 s of the CS). The pairings were separated by an interval of 100 s. Footshock sensitivity had been assessed, as described in Oriel et al. (2014), in adult mice pretreated postnatally with the same dose of CPF (or vehicle) in another unpublished study and was found not to differ amongst treatment groups. The apparatus was thoroughly cleaned with a dilute alcohol solution (35%) between each mouse. The order of testing among groups was random.
Day 3-Test 1: The mouse was placed in the test context 24 h after training. The cage was altered by placing a polypropylene sheet to cut off the corners thereby altering the contour of the cage and adding a drop of vanilla scent, thus ensuring that the tone cue was more salient than the contextual cue. Following 4 min acclimatization, 4 CS tones were presented, separated by intervals of 100 s. Freezing was scored blindly during the tones and the inter-tone intervals from videos, after first determining inter-observer reliability of two blind observers (*r* = 0.93).
Day 4-Extended training: After a 4 min habitation period, each mouse was exposed to 8 CS-US pairings as described above.
Day 5-Test 2: Each mouse was exposed to 4 CS tones in the altered context as described for Day 3.

### Active avoidance (AA)

Two-way AA testing was conducted in the same apparatus described in Section Apparatus, using both sides of the chamber. The day before the experiment, habituation to the apparatus and tones took place for 30 min, during which 5 tones 80 dB, 5 s duration, were presented at random intervals. Conditioning took place on 5 consecutive days with 30 trials per day. Thirty tones (20 s duration) served as the CS, with the footshock unconditioned stimulus (US 0.4 mA) delivered during the last 5 s of the CS. Three responses were measured automatically, as described previously (Levi et al., 2008): (1) Avoidance: move to the safe chamber after onset of the CS, but prior to the US, (2) Escape: move to the safe chamber only after US onset, (3) No response: Neither escape nor avoidance occurred (Levi et al., 2008). After each session, the apparatus was cleaned with a diluted solution of ethanol (35%) and water.

### Sweet preference

Mice were housed individually in cages that had two 200 ml bottles containing tap water for 5 days. For the next 6 days they had two bottles, one containing a 5% sucrose solution and the other tap water for 12 h in the dark phase, following 12 h deprivation in the light phase, switching the sides on day 3. Body weight was monitored weekly and fluid intake monitored daily. Sucrose preference was calculated as the percent of the total fluid intake.

### Forced Swim Test (FST)

One week after the sweet preference test, the mice were tested in the FST. Each mouse was filmed and scored blindly after first establishing reliability between two blind observers (*r* = 0.94) in the last 4 min as described in Lin et al. (2012). The dependent measures were: (1) Immobility: No movement except for minimal paw and tail movements necessary to keep afloat, (2) Climbing: rhythmic bilateral movements of forelimbs and hindlimbs with body stretched vertically along the periphery of the container, and (3) Swimming which was calculated as the difference between 4 min and the time spent in immobility plus climbing.

### Analysis of AChE variants gene expression using real-time PCR (RT-PCR)

The effect of CPF on the expression of AChE transcripts was assessed by quantitative RT-PCR analysis on the first and last injection days (PND-4 and −10) in frozen forebrains (rostral to the midbrain) that had been dissected 2 h after the last injection following isofluorane anesthesia.

In order to examine the long-term effect of CPF in adult mice on expression of mRNA of AChE splice variants, 2 h following the last testing session on Day 5 of the extended training, the brains of isofluorane anesthetized mice were removed, placed in ice-cold 0.9% saline. The frontal cortex, amygdala area, and hippocampus were dissected, frozen in liquid nitrogen, and stored at −80°C. Expression of AChE-R and AChE-S was analyzed by real time polymerase chain reaction (RT-PCR) (Dori et al., 2011). Total RNA was extracted and purified by using the RNeasy-mini kit of Qiagen. Essentially, the frozen brain samples were ruptured in 0.6 ml of lysis buffer using a 1.5 ml eppendorf tube-fit plastic pestle. The crude lysate was further homogenized by centrifugation through a QIA shredder spin column. In order to digest contaminated genomic DNA, the total RNA was treated with DNase-I according to the manufacturer's instructions. The quality of the total RNA was carried out using the Agilent RNA Nano Chip (Agilent Biotechnologies). Samples exhibiting RIN greater than 8.0 were used downstream. A non-coding cDNA strand was synthesized by using the Verso cDNA synthesis kit of ABgene® (Thermo Scientific) according to the manufacturer's instructions. One microgram of total RNA was used as template for the cDNA synthesis. A mixture (3:1) of random primers and anchored oligo-dT primers was employed. RT-PCR was used to detect the AChE splice variant expression in sample brains by using AbsoluteTM Blue qPCR ROX kit of ABgene® (Thermo Scientific) and Eco qPCR system (Illumina, San Diego, CA). The relative expression of each gene was calculated using the Pfaffl method (Pfaffl, 2001) implemented in the Eco qPCR system software. The mouse actin gene, which was similarly expressed in the samples (Dori et al., 2011; Ragu Varman et al., 2013), and was used following validation experiments as an internal vehicle in all RT-PCR experiments. A pool of all the samples was used for between-run normalization. The reactions were carried out in triplicate. The thermal cycling program was as follows: hold on 95°C for 15 min followed by 40 cycles of 10 s 95°C, 1 min 60°C. Supplementary Table 1 contains the primers' sequences for the genes examined and the respective efficiencies of their reactions. All primers are from PrimerDesign®. The sequence of β-actin is not noted due to intellectual property rights.

### Statistical analysis

All the data were analyzed using StatSoft STATISTICA software Version 9.0 (StatSoft, 2009), with significance at *p* < 0.05. All the values in figures and tables are presented as means and SEMs. Analyses of variance (ANOVA) were followed by *post-hoc* Duncan tests. For multiple testing days or age differences, data were analyzed by repeated measures ANOVA (RMANOVA) with age or day as the relevant repeated measure. The comparisons of interest were CPF vs. Vehicle for the effect of CPF and NT vs. Vehicle in order to examine the effect due to the stress of the injection. The variable for the effect of CPF treatment is designated as Treatment for pups and Pretreatment for adults.

## Results

### Body weight

Mouse pups did not show any overt signs of toxicity throughout the injection period. A 3-Way RMANOVA conducted between Treatment (NT, Vehicle, CPF), Strain (Balb/C; B6), and Day (PND-4 to -10) as a repeated measure, revealed the expected significant main effect of Day, *F*_(6, 792)_ = 634.8; *p* < 0.00001, indicating growth. The initial and end weight during the treatment period are summarized in Supplementary Table 2. In adult mice there were no significant difference in groups pretreated with CPF or vehicle, although there was a significant effect of Strain, *F*_(1, 83)_ = 28.94, *p* = 0.000001, indicating that Balb/C mice were significantly heavier than B6 mice.

### Brain acetylcholinesterase activity in pups

**Figure 2:**
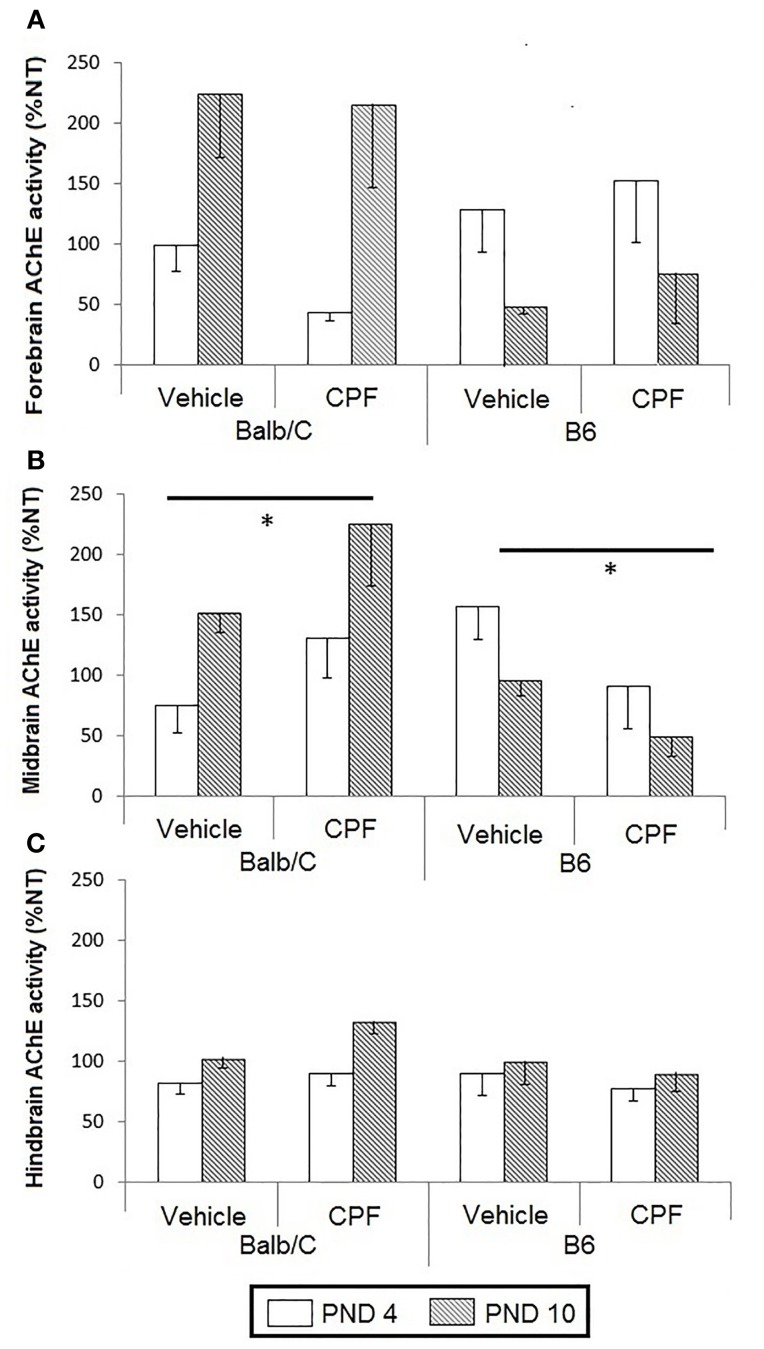
**AChE activity in pup brain expressed as percent of the corresponding NT group (Mean–SEM)**. **(A)** Forebrain, **(B)** Midbrain, **(C)**. Hindbrain. ^*^p < 0.01for the interaction between CPF treatment and strain. For each strain the effect of CPF differs from the NT group, *p* < 0.001. For clarity, effects not related to CPF treatment are not marked on the graph and are described in Section Brain Acetylcholinesterase Activity in Pups.

AChE activity in forebrain, midbrain, and hindbrain of pups was analyzed as the percent of activity of the NT groups by conducting a 3-Way ANOVA for the effect of Age (PND-4, PND-10), Treatment (Vehicle, CPF), and Strain (B6, Balb/C). A significant interaction between Treatment and Strain was found in the midbrain, *F*_(1, 38)_ = 8.33, *p* < 0.01. Analysis of this interaction revealed that although in the vehicle-treated groups, there was no difference between the strains, CPF elevated the activity of midbrain AChE in the Balb/C strain by 68%, while inhibiting AChE activity in the B6 strain by 65% (*p* = 0.001 for each contrast) (Figure 2).

Other strain and age differences between the groups were unrelated to CPF treatment. A significant Strain × Age interaction was found in both midbrain, *F*_(1, 38)_ = 10.56, *p* < 0.005, and in the forebrain, *F*_(1, 38)_ = 14.54, *p* < 0.0005. In both regions, the Balb/C mice showed increased AChE activity on PND 10 compared to PND-4 (*p* < 0.005 for both regions). AChE activity on PND-10, but not PND-4, was higher in the Balb/C strain compared to the B6 strain (*p* < 0.005 in both regions). In the hindbrain, there was a significant Age effect, *F*_(1, 41)_ = 5.27, *p* < 0.05, reflecting a relative increase in AChE activity in injected mice on PND-10 compared to PND-4, but there was no main effect or interaction involving CPF pretreatment (Figure 2).

### Pavlovian fear conditioning

Freezing and immobility were measured on Day 2, during the first 4 min prior to the training session, in order to examine if baseline activity of CPF-pretreated mice had been altered. No significant interaction was found for the effect of Pretreatment (CPF, Vehicle, and NT) × Strain (Balb/C; B6), *F*_(1, 46)_ = 1.23, n.s, or for the main effect of Pretreatment, *F*_(2, 46)_ = 1.21, n.s. However, a significant main effect of Strain was found, *F*_(1, 46)_ = 13.89, *p* = 0.0005. This main effect was illustrated by the chi-square distribution: χ^2^ = 22.05, *p* < 0.0001, demonstrating that C57 mice displayed immobility, whereas Balb/C mice showed more exploratory activity (Supplementary Figure 1) and Supplementary Method and Result (Contextual fear-conditioning exp.).

A 3-Way RMANOVA was conducted for the effects of Day, as a repeated measure (Day 3, Day 5), Strain (Balb/C, B6), and Pretreatment (CPF, Vehicle, NT) for the total freezing during the 4 tones and 4 inter-tone intervals. Following the analysis of total freezing, separate 3-Way RMANOVAs were run for the freezing during the tones and during the inter-tone-intervals to assess the effect of CPF on cued conditioning and generalization of fear.

#### Tones

There was a significant effect of Strain, *F*_(1, 43)_ = 19.82, *p* < 0.0001, indicating more freezing in the B6 mice, as reported previously (Oriel et al., 2014), the expected significant effect of Day, *F*_(1, 43)_ = 5.07, *p* < 0.05, indicating more freezing after the extended training session on Day 5, and a significant interaction between Pretreatment and Strain, *F*_(2, 43)_ = 3.78, *p* < 0.05. No other main effects or interactions were significant. The *post-hoc* Duncan test for relevant comparisons between CPF and Vehicle-treated groups and between NT and Vehicle-treated groups to test for the effect of stress did not reveal significant differences.

#### Intervals

**Figure 3:**
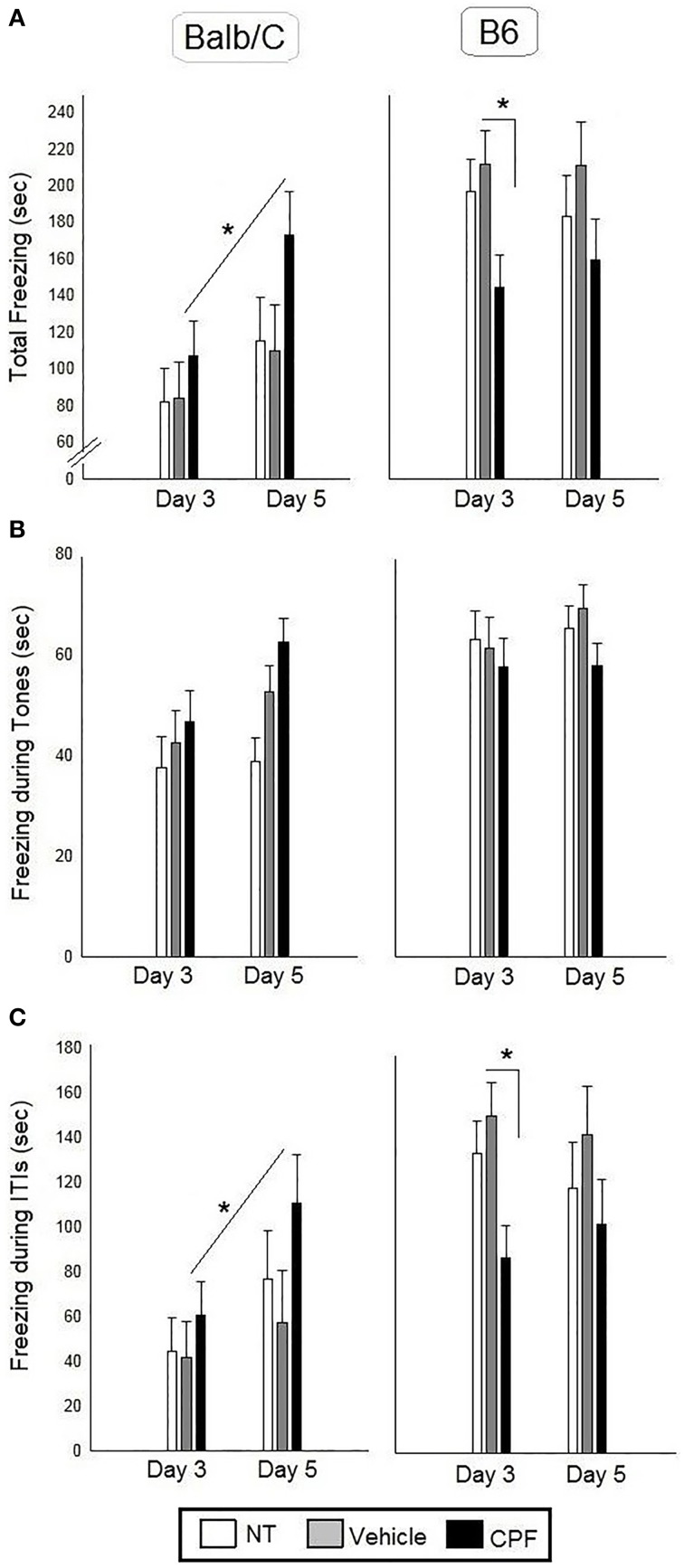
**Fear conditioning in adult Balb/C and B6 mice**. **(A)** Mean (± SEM) of freezing on Day 3 and on Day 5 [during the 4 tones and the 4 inter-tone intervals (ITIs)]. **(B)** Mean (± SEM) of freezing during the tones. **(C)** Mean (± SEM) of freezing during the ITIs. ^*^p < 0.05 CPF vs. Vehicle in Balb/C. For clarity, effects not related to CPF treatment are not marked on the graph and are described in Section Pavlovian Fear Conditioning. Balb/C: NT-N = 9; Vehicle-N = 7, CPF-N = 9 B6: NT-N = 9; Vehicle-N = 8, CPF-N = 9.

There was a significant effect of Strain, *F*_(1, 43)_ = 21.32, *p* < 0.00005, indicating more freezing in the B6 mice, a significant interaction between Pretreatment and Strain *F*_(2, 43)_ = 4.30, *p* < 0.05 and a significant interaction between Day and Strain, *F*_(1, 43)_ = 4.20, *p* < 0.05. No other main effects or interactions were significant. Duncan tests revealed that the B6-CPF mice showed significantly less freezing during the ITI's compared to B6-Vehicle mice (*p* < 0.05). The Day × Strain interaction indicated that the Balb/C mice showed significantly more freezing on Day 5 (extended training) compared to Day 3 (brief training) (*p* < 0.01), whereas B6 mice did not show any change with extended training (*p* = 0.81). The *post-hoc* analysis of the interaction between Day × Strain × Pretreatment revealed that only the CPF-treated Balb/C mice showed increased ITI freezing after extended training compared to brief training (*p* < 0.05), whereas the NT and Vehicle treated Balb/C groups did not show enhanced freezing (*p* = 0.17, *p* = 0.50, respectively) (Figure 3).

### Active avoidance

**Figure 4:**
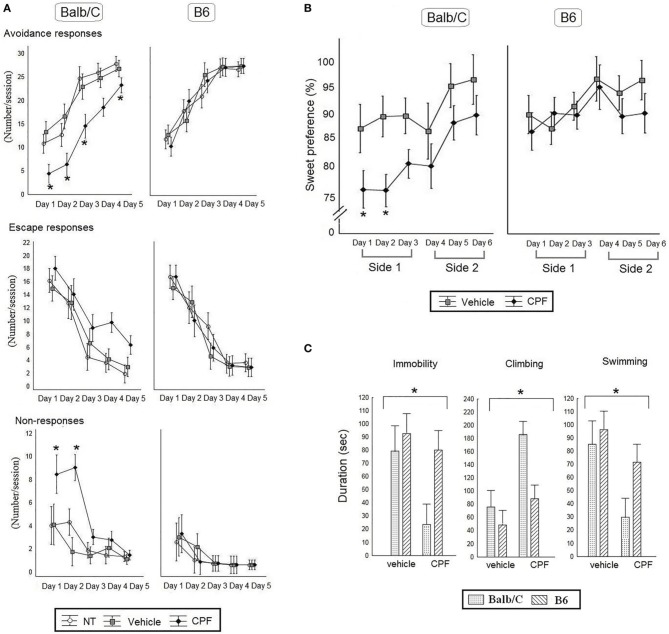
**(A)** Active Avoidance task in adult Balb/C and B6 mice. Mean (± SEM) of *Avoidance, Escape* and *No response*. ^*^p < 0.05 CPF vs. vehicle in Balb/C. *N* = 7 Balb/C- CPF mice and B6 vehicle and CPF mice, *N* = 6 Balb/C vehicle. **(B)** Percent preference for a sucrose solution [Mean (± SEM)] for 6 consecutive days. The side of the water and sucrose bottles was switched every 3 days (side 1 and side 2). ^*^p < 0.05 CPF vs. vehicle in Balb/C on day 1 and 2. **(C)** The average times (Mean (±) SEM) of immobility, climbing and swimming behaviors during the Forced Swim Test. **(B,C)**, *N* = 8 for all mice group except for *N* = 5 Balb/C Vehicle.

Active avoidance performance was assessed by analyzing the number of avoidance responses, escape responses, and non-responses by 3-Way RMANOVA for the effect of Day, Pretreatment, and Strain. Learning of the active avoidance response was confirmed by a main effect of Day, confirming increased avoidance over time, *F*_(4, 140)_ = 119.76, *p* < 0.000001), and the concomitant decrease in escape responses over days, *F*_(4, 140)_ = 77.65, *p* < 0.000001). A significant 2-Way interaction between Strain × Pretreatment, *F*_(2, 35)_ = 3.64, *p* < 0.05 for avoidance responses was further analyzed by *post-hoc* Duncan's test, indicating that in the Balb/C strain CPF-pretreated mice showed significantly less avoidance compared to the Vehicle and NT control groups (*p* < 0.01 in each comparison). CPF pretreatment did not affect the number of escape responses (Figure 4A). The lack of a CPF effect on escape responses, despite the obvious impairment in avoidance, suggested that the CPF-pretreated mice failed to react appropriately to the shock. The ANOVA for the effect of Strain × Day × Pretreatment for the non-responses revealed a significant three-way interaction, *F*_(8, 140)_ = 2.14, *p* < 0.05). *Post-hoc* Duncan analysis revealed that only the Balb/C mice pretreated with CPF showed a significant number of non-responses on Days 1 and 2. The high level of non-response in CPF-pretreated mice on the AA test is reminiscent of helplessness. Consequently anhedonia and FST tests, two standard assays of depression, were administered in a separate cohort.

### Sweet taste preference

A 3-Way ANOVA was conducted for the effect of Pretreatment (Vehicle, CPF), Strain (Balb/C, B6), and Day as a within-subject variable and Weight as a covariate, on the percentage of sucrose solution intake. A significant main effect of Pretreatment, *F*_(1, 25)_ = 6.87, *p* = 0.02, indicated that CPF-pretreated mice showed less sweet preference compared to the vehicle-pretreated mice. Balb/C mice consistently showed a lower preference for sucrose solution compared to B6 mice, leading to a main effect of Strain, *F*_(1, 25)_ = 5.96, *p* = 0.02. As expected there was a significant main effect of Day *F*_(5, 125)_ = 4.17, *p* = 0.002, showing increased sucrose consumption over time. None of the interactions was found significant (Figure 4B).

### Forced swim test

Immobility, climbing and swimming in the FST during the last 4 min of the 6 min exposure were analyzed first by MANOVA, which showed significant effects for Pretreatment and Strain (Wilk's lambda = 4.89, 4.19, respectively, *p* < 0.05) and then each variable was analyzed separately by 2-Way ANOVA for the effect of Pretreatment and Strain. CPF reduced immobility, *F*_(1, 25)_ = 4.40, *p* < 0.05 and swimming, *F*_(1, 25)_ = 7.05, *p* < 0.05, and significantly enhanced climbing, *F*_(1, 25)_ = 11.80, *p* < 0.005. There was also a significant effect of Strain, such that the Balb/C mice showed less immobility, *F*_(1, 25)_ = 4.51, *p* < 0.05, and increased climbing *F*_(1, 25)_ = 7.86, *p* < 0.01, compared to the B6 mice but the interaction between Strain and Pretreatment was not significant, *F*_(1, 25)_ = 2.82, *p* = 0.10 (Figure 4C).

### Expression of AChE splice variants in Balb/C and B6 pups

The immediate effect of CPF on PND-4 (the first day of the injection) and on PND-10 (the seventh day of injections) on the expression of AChE splice transcript, was assessed by quantitative RT-PCR analysis. A 3-Way RMANOVA for the effect of Treatment × Strain × Age was conducted separately for AChE-R and AChE-S transcripts. A significant effect of Age, *F*_(1, 68)_ = 6.48, *p* < 0.01, and a significant interaction between Age and Treatment, *F*_(2, 68)_ = 3.48, *p* < 0.05, were found for expression of AChE-R mRNA. The Duncan test revealed that the expression of AChE-R transcript was lower on PND-10 than on PND-4 for the Vehicle and CPF groups but not for the NT group. On PND-10 the Vehicle control group showed greater expression of AChE-R transcripts than the NT group, suggesting that injection stress enhanced the age-related escalation in AChE-R transcript expression in the forebrain. Similarly, expression of AChE-S transcripts was affected by Age, *F*_(1, 68)_ = 71.86, *p* < 0.00001, indicating higher expression of AChE-S transcripts on PND-10 compared to PND-4 and by Strain, *F*_(1, 68)_ = 7.80, *p* < 0.01, indicating higher expression of AChE-S transcripts in the B6 strain. Although these effects were modified by a 3-way interaction between Age, Strain, and Treatment, *F*_(2, 68)_ = 4.24, *p* < 0.05, *post-hoc* comparisons did not reveal specific effects of stress (NT vs. Vehicle) or of CPF (Vehicle vs. CPF) in either strain (Table 1).

**Table 1:** **Expression of AChE-R and AChE-S transcripts in forebrain of mouse pups**.

**Strain**	**Age**	**Treatment (N)**	**AChE-R**		**AChE-S**	
			**Mean**	**SEM**	**Mean**	**SEM**
Balb/C		NT (6)	1.13	0.13	1.11	0.17
	PND-4	Vehicle (7)	1.40	0.17	0.97	0.10
		CPF (7)	1.10	0.18	0.96	0.11
		NT (7)	0.96	0.15	1.45	0.08
	PND-10	Vehicle (6)	0.62	0.09	1.75	0.16
		CPF (6)	0.95	0.22	1.46	0.21
B6		NT (7)	1.03	0.07	1.09	0.09
	PND-4	Vehicle (7)	1.12	0.08	1.29	0.21
		CPF (8)	1.30	0.10	0.93	0.06
		NT (7)	1.41	0.45	2.02	0.12
	PND-10	Vehicle (6)	0.91	0.08	1.69	0.18
		CPF (5)	0.76	0.17	2.01	0.10

### Expression of AChE splice variants following cued fear conditioned in adult mice

Analysis of the expression of AChE splice transcripts in relevant brain regions (frontal cortex, hippocampus, amygdala) was carried out by RT-PCR following the 24 h retention test of the extended training session (Day 5). Each brain region was analyzed separately by 2-Way ANOVA for the effect of Strain and Pretreatment on each transcript. There was no main effect of Pretreatment or Strain and no interaction for the expression of AChE-R mRNA in the frontal cortex. There was a main effect of Strain for the expression of AChE-S transcript in the frontal cortex, *F*_(1, 40)_ = 5.50, *p* < 0.05, indicating higher expression of AChE-S transcripts in the B6 strain, similar to the difference that was revealed in the forebrain of B6 pups.

In the hippocampus, there was a main effect of Strain for the expression of AChE-R, *F*_(1, 42)_ = 10.72, *p* < 0.005, indicating higher expression of AChE-R transcripts in the Balb/C strain. Since we had an a priori hypothesis (Nijholt et al., 2004; Oriel et al., 2014) that the CPF-treated Balb/C mice would have greater expression of AChE-R, we probed the difference between the CPF and Vehicle treated mice in the Balb/C strain using in a *post-hoc* comparison, which confirmed that CPF-treated mice in the Balb/C strain had higher expression of AChE-R than Vehicle-treated mice (*p* < 0.05). A similar analysis of the AChE-S transcript revealed a significant effect of Pretreatment, *F*_(2, 42)_ = 5.64, *p* < 0.01, indicating greater expression of AChE-S transcripts in the hippocampus of CPF-pretreated Balb/C mice compared to Vehicle-treated Balb/C mice (*p* = 0.003). No effect of CPF was found in the B6 strain.

**Figure 5:**
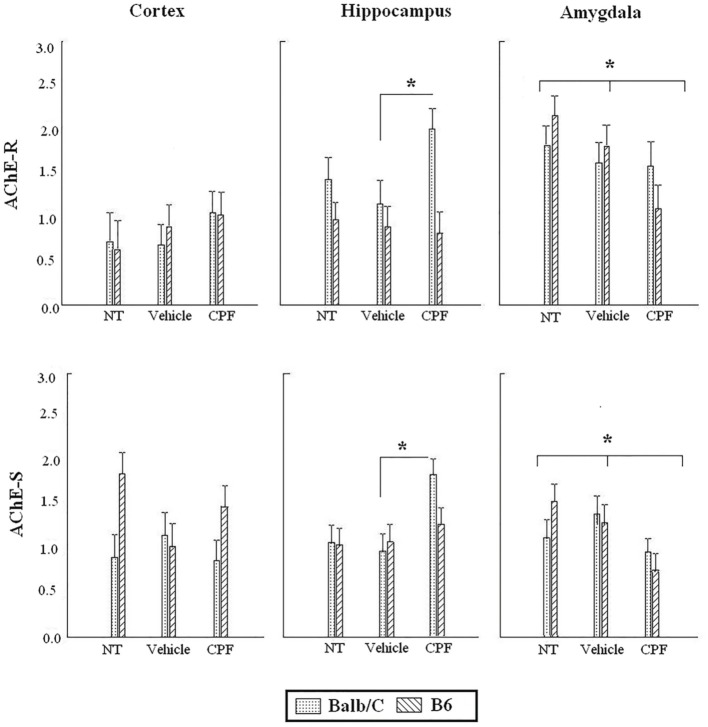
**Expression of AChE-R and AChE-S in adult brain regions following cued fear conditioning**. Data are mean (±)SEM of AChE-R or AChE-S splice variants in the frontal cortex, hippocampus and amygdala. ^*^p < 0.05 CPF vs. Vehicle in Balb/C in the hippocampus. ^*^p < 0.05 CPF vs. NT in the amygdala of B6 and Balb/C mice. *N* = 8–9 for all CPF groups. *N* = 7–9 for B6 vehicle and NT groups. *N* = 6–8 for Balb/C vehicle and NT groups.

In the amygdala, a significant reduction in expression of both transcripts was found in mice treated with CPF. There was significantly less AChE-R transcript expression, *F*_(2, 43)_ = 3.53, *p* < 0.05, in mice treated with CPF compared to NT (*p* = 0.01) and a significantly lower expression of AChE-S transcripts in the amygdala of mice treated with CPF, *F*_(2, 45)_ = 4.96, *p* = 0.01, compared to both NT and Vehicle pretreated groups (Figure 5).

## Discussion

Repeated developmental exposure to CPF caused long-term effects on emotional behavior in Balb/C and B6 mice, which were more pronounced in the Balb/C strain. Adult Balb/C mice pretreated with CPF showed enhanced fear-conditioning, whereas B6 mice exposed to CPF postnatally, showed better tone-specific discrimination than Vehicle-treated mice, suggesting that they learned to regulate their fear response, limiting it to the cue.

The difference in emotional reaction to postnatal CPF may reflect known strain differences (Anisman et al., 1998; Belzung and Griebel, 2001) as Balb/C mice show more context-induced freezing (a possible parallel to inter-tone intervals) than B6 mice (Diamantopoulou et al., 2012). Fear conditioning serves as a model for the underlying pathology of anxiety disorders, including post-traumatic stress disorder and panic attacks (Maren, 2008). The enhanced fear-conditioning in CPF-pretreated Balb/C mice (current study) and in both Balb/C and B6 mice exposed postnatally to DFP (Oriel et al., 2014), suggests that early OP exposure may enhance vulnerability to anxiety or stressor-related disorders in adults. The strain difference in reaction to emotional behavior might be related to the marked strain difference in AChE enzyme activity in the pups following the daily injections. Induction of plasma AChE was reported weeks after administration of CPF and CPF oxon at doses several fold higher than used in the current study (Chiappa et al., 1995; Duysen and Lockridge, 2011). In addition, increased AChE activity was observed in brain following exposure to malathion and sarin (Trevisan et al., 2008; Bansal et al., 2009). In the present study the Balb/C strain showed greater AChE activity in the forebrain and midbrain in wake of the daily injections, but only in the midbrain was there a clear effect of CPF to increase AChE activity in the Balb/C strain and inhibit activity in the B6 strain relative to the NT group. Given the role of AChE non-catalytic effects in the developing brain (Day and Greenfield, 2002), further research is required to explore whether changes in pup brain AChE activity are related to changes in sensitivity to emotional behavior observed in pre-treated adult mice.

In addition to the marked strain difference in fear-conditioning, postnatal CPF led to behaviors that resemble an agitated form of depression (Akiskal et al., 2005), namely anhedonia and increased climbing behavior, rather than immobility, in the forced swim test. The effects were more pronounced in the Balb/C strain, although the strain by pretreatment interactions did not reach statistical significance. Similarly, anhedonia was found in Sprague–Dawley rats following postnatal CPF or diazinon on postnatal days PND 1–4 (Aldridge et al., 2005a; Roegge et al., 2008). Depression may be characterized by concurrent symptoms of opposite polarity such as anhedonia and psychomotor agitation and goal-directed behavior (Akiskal and Benazzi, 2005; Akiskal et al., 2005). Climbing behaviors were found in depressed rats treated with reboxetine, a norepinephrine uptake inhibitor (Detke et al., 1995) and in dopamine transporter (DAT) null mice (Perona et al., 2008). CPF and its active metabolite CPF-oxon were found to affect monoaminergic transmission (Slotkin et al., 2002; Aldridge et al., 2005a,b), which could enhance the risk for depressive behavior (Stallones and Beseler, 2002). Anhedonia and enhanced climbing were more salient in the Balb/C strain which also showed helplessness by failing to escape or avoid footshock in the active avoidance task.

Despite the fact, that low dose of CPF did not alter expression of AChE transcripts and did not significantly inhibit AChE activity in pups, upon maturation significant long-term effects of CPF were observed in the expression of AChE mRNA in the hippocampus and amygdala, revealing a delayed treatment effect at adulthood. CPF-pretreated adult Balb/C mice showed higher expression of AChE-R and AChE-S in the hippocampus. In contrast, the expression of AChE-S transcripts in the amygdala was reduced by postnatal CPF. Since hippocampal projections to amygdala modulate conditioned freezing (Sierra-Mercado et al., 2011), the reduced expression of AChES mRNA following CPF in the amygdala and higher expression of both splice variants in the hippocampus might be related to the changes in conditioned fear behavior. Although a previous report showed that neither AChE-R nor AChE-S mRNA expression was altered in the forebrain or brainstem of adults treated with postnatal CPF, enhanced expression was found *ex vivo* after postnatal diazinon and *in vitro* following both of these OPs (Jameson et al., 2007). The current study, which specifically localized changes in AChE transcript expression to two major limbic structures involved in regulating the behavioral response to stress, supports the role of altered AChE transcript expression in the delayed exacerbation of anxiety and depression.

In conclusion, enhanced expression of hippocampal AChE transcripts in fear-conditioned mice and exacerbation of conditioned fear resulted from postnatal exposure to both CPF (current study) and DFP (Oriel et al., 2014), alerting researchers to the probability that OP exposure can enhance the vulnerability of exposed children to pathological emotional reactions in the face of adverse life events.

## Author contributions

OK and SO designed the study, analyzed the data and wrote the manuscript. SO performed the experiments.

### Conflict of interest statement

The authors declare that the research was conducted in the absence of any commercial or financial relationships that could be construed as a potential conflict of interest.

## Abbreviations

CPF: chlorpyrifos
PND: postnatal day
OP: organophosphate pesticide
AChE: acetylcholinesterase
AChE-R: Read-through AChE
AChE-S: Synaptic AChE
B6: C57Bl/6
AA: active avoidance
FST: Forced swim test, ITI, inter-trial intervals.
