# Supplementary material for: Strain dependent effects of conditioned fear in adult C57Bl/6 and Balb/C mice following postnatal exposure to chlorpyrifos: relation to expression of brain acetylcholinesterase mRNA
Source: Front Behav Neurosci. 2015 Apr 29;9:110. doi: 10.3389/fnbeh.2015.00110 (PMC4413781; doi:10.3389/fnbeh.2015.00110)
Supplement: Supplementary file 1 [file Table1.DOCX]

***Supplementary Material***

**Strain dependent effects of conditioned fear in adult C57Bl/6 and Balb/C mice following postnatal exposure to chlorpyrifos: Relation to expression of brain acetylcholinesterase mRNA**

**Sarit Oriel^1^, Ora Kofman^1^***

^1^ Department of Psychology and Zlotowski Center for Neuroscience, Ben-Gurion University of the Negev, Beer-Sheva, IL 84105, Israel.

*** Correspondence:** Ora Kofman, Zlotowski Center for Neuroscience, Department of Psychology, Ben-Gurion University of the Negev, P.O.B. 653, Beer-Sheva, IL 84105, Israel.

kofman@bgu.ac.il (O. Kofman).

## Supplementary Tables

**Supplementary Table 1.** Primers for AChE splice variant used for RT-PCR analyses.

| **Gene** |  | **Sequence** | **Efficiency** |
| --- | --- | --- | --- |
| **AChE-R** | Sense Primer | E4 (648(+)GGAGCAGGGAATGCACAAG) | 100.3 |
|  | Anti-sense Primer | I4 (741(-)TGGGGAGGTGGAGAAGAGAG) |  |
| **AChE-S** | Sense Primer | E4(1744(+)CTGAACCTGAAGCCCTTAGAG) | 97.4 |
|  | Anti-sense Primer | E6 (1855(-) CCGCCTCGTCCAGAGTATC) |  |
| **β-actin** |  |  | 87.3 |
|  |  |  |  |
