# Supplementary material for: Strain dependent effects of conditioned fear in adult C57Bl/6 and Balb/C mice following postnatal exposure to chlorpyrifos: relation to expression of brain acetylcholinesterase mRNA
Source: Front Behav Neurosci. 2015 Apr 29;9:110. doi: 10.3389/fnbeh.2015.00110 (PMC4413781; doi:10.3389/fnbeh.2015.00110)
Supplement: Supplementary file 2 [file Table2.DOCX]

***Supplementary Material***

**Strain dependent effects of conditioned fear in adult C57Bl/6 and Balb/C mice following postnatal exposure to chlorpyrifos: Relation to expression of brain acetylcholinesterase mRNA**

**Sarit Oriel^1^, Ora Kofman^1^***

^1^ Department of Psychology and Zlotowski Center for Neuroscience, Ben-Gurion University of the Negev, Beer-Sheva, IL 84105, Israel.

*** Correspondence:** Ora Kofman, Zlotowski Center for Neuroscience, Department of Psychology, Ben-Gurion University of the Negev, P.O.B. 653, Beer-Sheva, IL 84105, Israel.

kofman@bgu.ac.il (O. Kofman).

## Supplementary Tables

**Supplementary Table 2.** Body weight in grams at PND-4 and PND-10.

|  |  |  | **PND-4** | | **PND-10** | | **Adult** | |
| --- | --- | --- | --- | --- | --- | --- | --- | --- |
| **Strain** | **Treatment** | **N (litters)** | Mean | SEM | Mean | SEM | Mean | SEM |
| **BALB/C** | NT | 17 | 3.04 | 0.18 | 6.45 | 0.24 | 30.98 | 0.9 |
|  | control | 28 | 2.92 | 0.12 | 5.96 | 0.30 | 29.42 | 0.48 |
|  | CPF | 29 | 3.12 | 0.12 | 6.25 | 0.23 | 29.98 | 0.8 |
| **C57** | NT | 16 | 3.00 | 0.14 | 6.12 | 0.31 | 27.48 | 0.4 |
|  | control | 23 | 3.12 | 0.10 | 6.00 | 0.22 | 26.97 | 0.4 |
|  | CPF | 25 | 2.95 | 0.11 | 5.78 | 0.24 | 27.7 | 0.5 |
