# Supplementary material for: Strain dependent effects of conditioned fear in adult C57Bl/6 and Balb/C mice following postnatal exposure to chlorpyrifos: relation to expression of brain acetylcholinesterase mRNA
Source: Front Behav Neurosci. 2015 Apr 29;9:110. doi: 10.3389/fnbeh.2015.00110 (PMC4413781; doi:10.3389/fnbeh.2015.00110)
Supplement: Supplementary file 3 [file DataSheet1.DOCX]

***Supplementary Material***

**Strain dependent effects of conditioned fear in adult C57Bl/6 and Balb/C mice following postnatal exposure to chlorpyrifos: Relation to expression of brain acetylcholinesterase mRNA**

**Sarit Oriel^1^, Ora Kofman^1^***

^1^ Department of Psychology and Zlotowski Center for Neuroscience, Ben-Gurion University of the Negev, Beer-Sheva, IL 84105, Israel.

*** Correspondence:** Ora Kofman, Zlotowski Center for Neuroscience, Department of Psychology, Ben-Gurion University of the Negev, P.O.B. 653, Beer-Sheva, IL 84105, Israel.

kofman@bgu.ac.il (O. Kofman).

## Supplementary Figure

Baseline level: Mean (+SEM) of freezing on Day 2, prior to the training. A significant main effect of Strain was detected and represented by * p=.0005.

Balb/C: NT-N= 9; Vehicle-N=7, CPF-N=9

B6: NT-N= 9; Vehicle-N=8, CPF-N=9.
