# Supplementary figures and images for: Strain dependent effects of conditioned fear in adult C57Bl/6 and Balb/C mice following postnatal exposure to chlorpyrifos: relation to expression of brain acetylcholinesterase mRNA
Source: Front Behav Neurosci. 2015 Apr 29;9:110. doi: 10.3389/fnbeh.2015.00110 (PMC4413781; doi:10.3389/fnbeh.2015.00110)

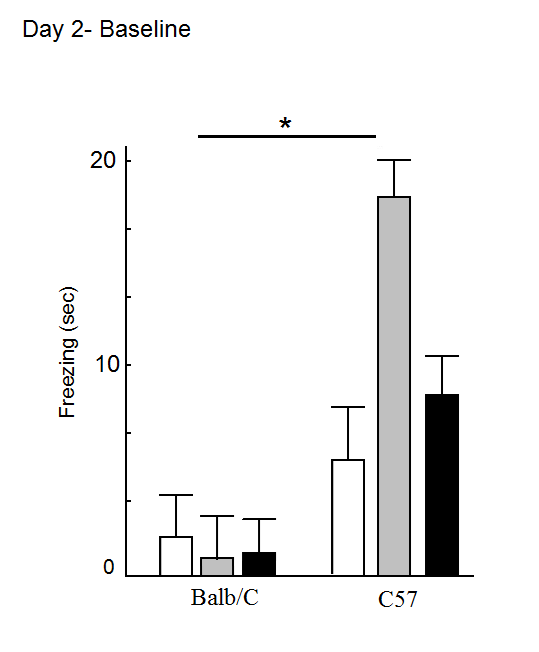

Supplement: Supplementary file 4 [file Image1.PNG]
